# Extrapyramidal Symptoms in a Bipolar 1 Patient Following Re-initiation of Lithium: A Case Report

**DOI:** 10.7759/cureus.39361

**Published:** 2023-05-22

**Authors:** Godwin I Orji, Maria Mansoor, Sophia B Bellegarde, Lauren Punter, Nkolika Odenigbo, Patrice Fouron

**Affiliations:** 1 Psychiatry, Interfaith Medical Center, New York, USA; 2 Psychiatry, American University of Antigua, Coolidge, ATG

**Keywords:** amantadine, cogwheel rigidity, extrapyramidal, bipolar disorder, lithium

## Abstract

A Chinese American female presents with extrapyramidal symptoms following re-initiation of lithium carbonate as part of her treatment plan following a recent manic episode. She developed neurological symptoms including stiffness in the upper extremities and restlessness shortly after the medication was initiated. The patient has a history of bipolar disorder for which she has been treated with lithium carbonate among other mood stabilizers, but the patient was not compliant with medications. Though rare in presentation, this case report aims to highlight the importance for clinicians to recognize the possibility of extrapyramidal symptoms developing in patients taking lithium regardless of the duration of the lithium therapy.

## Introduction

Lithium is a commonly used mood stabilizer for the treatment of bipolar disorder [1]. Lithium has been found to be effective in the management of acute manic and depressive episodes, as well as in minimizing the recurrence of mood episodes and reducing the risk of suicidal behaviors [1,2]. This psychotropic agent has extensively been studied since its use in psychiatry for over 60 years. The mechanism by which it exerts its therapeutic effects is still not fully understood [3,4]. Lithium is said to inhibit two enzymatic pathways: inositol monophosphatase within the phosphatidylinositol signaling pathway, and the protein kinase glycogen synthase kinase [1,3,5]. It also decreases excitatory neurotransmission at the cellular level by lowering dopamine and glutamate levels. It increases inhibitory transmission by increasing GABA and serotonin levels. Lithium also downregulates N-methyl D-aspartate receptors [5]. Lithium is excreted almost entirely (95%) by the kidney with only a small amount lost with feces and sweat [6]. Common side effects found in patients being treated with lithium include dyspepsia, nausea, vomiting, and diarrhea. Patients have also complained of weight gain, increased thirst, polyuria, as well as acne upon initiation of treatment [6]. Lithium can independently cause extrapyramidal symptoms (EPS) in a minority of patients and is also known to worsen neuroleptic-induced EPS [7,8]. The first documented report of EPS following lithium use was published in 1975 in a study of patients receiving lithium for maintenance treatment of bipolar disorder and schizoaffective disorder. The study showed a positive correlation between the development of EPS and the duration of lithium use [9]. The study also showed that the extrapyramidal side effects seen with lithium use are not treatable with benztropine as compared to those seen with antipsychotics [9]. Another study showed no systematic relationship between the development of EPS and the duration of lithium therapy [10]. Thus far, there remains a dearth of literature to support EPS like cogwheel rigidity, muscle stiffness, and restlessness as adverse outcomes of lithium therapy. Hence, we present a case of extrapyramidal side effects occurring during treatment with lithium in a patient with bipolar 1 disorder with reported comorbid generalized anxiety disorder, which resolved a few days after changing medication to another mood stabilizer.

## Case presentation

The patient is a 29-year-old Chinese American female, single, unemployed, domiciled with parents and brother with a reported past psychiatry history of bipolar 1 disorder and generalized anxiety disorder and no significant non-psychiatric medical history. The patient was brought by Emergency Medical Services which was activated by her brother due to disorganized and aggressive behavior. On evaluation in the psychiatry emergency department (ED), she reported a euphoric mood, decreased need for sleep, reported sleeping only 2-3 hours per night over the past three days and still had a lot of energy. She reported spending a lot of money recently on items she does not need. She reported being in love with a South Korean male model on Instagram whom she communicates via telepathic means. She also exhibited grandiose delusion of having special powers and communicating with God. Her speech was rapid and pressured. Her affect was labile and expansive. The patient was hyperactive and pacing the emergency room. The patient has had five previous hospitalizations for manic episodes since 2016 with the most recent hospitalization a few months prior to the current presentation. Past medication trials include lithium, aripiprazole, risperidone, and lumateperone. She was not compliant with medications reporting that she has not taken medications for months. Laboratory investigations including complete blood count (CBC) and comprehensive metabolic panel (CMP) were within normal range. Chest X-ray was normal. The patient, however, tested positive for COVID-19 but was asymptomatic. The patient was admitted and requested to be restarted on lithium as it had helped her in the past. She was started on lithium 300 mg twice daily. Trough lithium levels measured after five days was 0.3 mEq/L. Lithium was increased to lithium 300 mg three times daily. Two days following the initiation of lithium, the patient is noted to have some stiffness in her arms and neck, cogwheel rigidity, coarse tremors of the upper limbs, and hypokinesia on physical examination. Following an increase in the dose of lithium, the severity of stiffness and cogwheel rigidity increased with associated restlessness, dysphagia, and hypersalivation. The patient was started on benztropine 2 mg IM once and 1 mg per oral twice daily for the EPS and glycopyrrolate 0.1 mg IM once then 2 mg per oral three times daily as needed for the hypersalivation. The hypersalivation resolved by day 8; however, the patient continued to experience rigidity in her arms and neck. Lithium was discontinued on day 12. The EPS started to resolve in the days following the discontinuation of the lithium with eventual complete resolutions of symptoms. The patient was started on quetiapine IR 25 mg at bedtime and oxcarbazepine 300 mg twice daily for mood stabilization with a resolution of her manic symptoms. The patient's delusions of being in a relationship with a famous South Korean model remained and she was discharged with an aftercare appointment for outpatient follow-up. 

## Discussion

Lithium is one of the most studied psychotropic agents used in psychiatry for the treatment of bipolar spectrum disorders and its benefits in the treatment of bipolar disorders and its anti-suicidal activity are well established [11]. Common side effects seen with lithium therapy include gastrointestinal symptoms such as dyspepsia, nausea, vomiting, and diarrhea as well as weight gain, increased thirst, tremor, sedation, acne, and decreased cognition [12]. EPS induced by lithium treatment are rare but have been reported by multiple authors [13-17]. Lithium has also been reported to potentiate and exacerbate the EPS effects of antipsychotics [13,15]. Our case report shows evidence of lithium producing the extrapyramidal side effects of cogwheel rigidity, stiffness, and coarse tremors as the patient improved following the discontinuation of the lithium. There was no recurrence of the EPS following the administration of oxcarbazepine and quetiapine. The mechanism by which lithium induces EPS is not exactly known but there have been reports of selective dopamine blockade by lithium [13]. Past reports of EPS in patients on lithium have mostly been in patients with chronic use of lithium, toxic or close to toxic levels of lithium [15,17]. However, as seen in our patient, these adverse effects can occur in patients with the recent initiation of lithium therapy and with normal or subtherapeutic lithium levels. Lithium-induced EPS can also occur in patients with past tolerance to lithium use as seen in our patient who had used lithium in the past without developing similar EPS effects. Management of lithium-induced EPS is similar to that caused by antipsychotics including the use of anticholinergics like benztropine and discontinuation of the implicated compound. However, there have been reports that the motor disturbance seen in lithium-induced EPS is not relieved by anticholinergics such as benztropine [16]. The rigidity seen in our patient did not resolve with the administration of benztropine despite adequate trial for about seven days until lithium was discontinued. Amantadine has been reported to improve lithium-induced EPS compared to anticholinergic agents like benztropine and diphenhydramine; however, amantadine was not tried in our patient [16].

## Conclusions

In this case, report we document the development of EPS following the re-initiation of lithium with resolution only after discontinuation of lithium. This case report adds to a collection of case reports documenting this rare side effect of lithium. EPS can also be seen with low serum levels of lithium and benztropine may not be effective in the treatment of EPS induced by lithium. Our case report supports that clinicians should consider lithium as a possible cause of EPS even if the patient has just started the medication or has normal or low serum levels of lithium. 
